# Prevalence and factors associated with potential drug-drug interactions in prescriptions presented at private pharmacies in Mbarara city, southwestern Uganda

**DOI:** 10.1186/s40360-023-00719-1

**Published:** 2024-01-02

**Authors:** Allan Phillip Lule, Ogwal Basil Delic, Keneth Katunguka, Francis Muwonge, Tadele Mekuriya Yadesa

**Affiliations:** 1 Department of Pharmacy, Faculty of Medicine, Mbarara University of Science and Technology, P.O.Box 1410, Mbarara, Uganda; 2 Department of Clinical Pharmacy and Pharmacy Practice, Kampala International University, Ishaka, Uganda

**Keywords:** Prevalence, Potential drug − drug interactions, Risk factors, Mbarara, Uganda

## Abstract

**Background:**

Drug-drug interactions (DDIs) influence the effectiveness of medication and thus determine the treatment outcomes of diseases managed with pharmacotherapy. This study aimed to determine the prevalence, severity, and factors associated with potential drug-drug interactions in prescriptions presented at private pharmacies in Mbarara city.

**Methods:**

DDIs were identified and classified basing on risk and severity using Lexicomp drug interaction database. STATA version 13 was used to analyze the collected data. Descriptive statistics were used to summarize the severity of potential DDIs identified. Bivariate and multivariate logistic regression was employed to identify different factors associated with the presence of potential DDIs.

**Results:**

A total of 295 prescriptions from 18 private pharmacies were studied and the prevalence of clinically significant potential DDIs was 37.6%. About half (149, 50.5%) of the patients were females, the majority (199, 67.5%) were adults 18–59 years of age whereas most (208, 70.5%) had a comorbid condition. Over one half (162, 54.9%) of the prescriptions were received from hospitals and majority of the prescriptions had 4 drugs prescribed (*n* = 175, 59.32%). Having one or more comorbidities and prescribing of therapeutic drug categories including anti-fungal, antihypertensives, analgesics, or corticosteroids were significantly associated with potential DDIs.

**Conclusions:**

The prevalence of potential drug-drug interactions in outpatient setting in Mbarara city was high and majority of the potential DDIs were of moderate severity. Having 1 or more comorbidities and prescribing of therapeutic drug categories including antifungals, antihypertensives, analgesics, or corticosteroids were significantly associated with potential DDIs.

## Background

A drug-drug interaction (DDI) is defined as a pharmacokinetic or pharmacodynamic influence of drugs on each other, which may result in undesired effects such as reduced effectiveness or increased toxicity [1]. According to the Food and Drug Authority, drug-drug interactions occur when two or more drugs react with each other and this may make the interacting drugs less effective, cause unexpected side effects or increase the action of a particular drug [2]. Different drugs are often used concurrently with others to achieve a desired therapeutic goal and treat coexisting diseases or multi-morbidity conditions [3]. The presence of multi-morbidity increases the possibility of patients using many drugs concurrently, and this is associated with a high risk of potential drug-drug interactions [4, 5]. The concurrent use of many drugs (4 or more) is referred to as polypharmacy [6].

DDIs can be classified into two main groups i.e., pharmacokinetic DDIs and pharmacodynamic DDIs. Pharmacokinetic DDIs involve interference with the absorption, distribution, metabolism and excretion of one drug by another, whereas pharmacodynamic DDIs may be due to: direct effect of a drug on function of the receptor of another, interference with a biological or physiological control process and additive/opposed pharmacological effect of one drug on another. The toxic effects of DDIs may be adverse drug events that can be severe enough to necessitate hospitalization and increase hospital stay. Drug-drug interactions may also lead to poor health outcomes, as well as increased costs and utilization of healthcare service [7, 8].

In all health care systems, there is need for information on the occurrence of potential DDIs, knowledge of their associated factors and tools to identify them. These assist prescribers and other members of healthcare teams in predicting, identifying, and managing potential drug-drug interactions so as to minimize damages caused by them [9].

There are several factors that contribute to the occurrence of potential DDIs, including the number of medications prescribed at any given time, therapeutic drug classes in prescriptions, the patient’s gender, age, number of prescribers involved, the presence of comorbidities, coinfections, and the availability of potential DDI identification tools [10].

From real-world data, there is proof of high prevalence of potential drug-drug interactions worldwide. Nearly 74,000 emergency room visits and 195,000 hospitalizations in the USA every year are caused by drug-drug interactions, resulting from the ineffectiveness of current approaches to DDI identification [11, 12].

Similarly, overall prevalence of potential DDIs in developing countries is high, ranging from 23 to 86% according to reports from hospital settings in Uganda, Ethiopia, Pakistan and Iran. Additionally, Uganda’s capital Kampala had a high overall prevalence of potential DDIs of 89.3% among hospitalized HIV patients presenting with suspected meningitis in 2020 [13]. According to a study conducted by Lubinga and Uwiduhaye in 2011, 23% of in-patient prescriptions in Mbarara Regional Referral Hospital had potential DDIs [7]. A recent cross-sectional study conducted in the oncology unit of the same hospital revealed a 60.3% prevalence of clinically significant DDIs among chemotherapy patients, majority of whom (79.9%) were outpatients [14]. To the best of our knowledge, there is no country wide study that explored the prevalence of potential DDIs in Uganda.

Shortage of information concerning DDIs in resource-limited countries like Uganda results in more DDI related damage than in developed countries [15]. Despite understanding the role played by DDIs in therapeutic outcomes, there was limited information regarding their prevalence in outpatient settings in Mbarara, Southwestern Uganda and Uganda at large. At the time of this study, the prevalence of potential DDIs in Southwestern Uganda had last been documented in a 2008 study on inpatients at Mbarara Regional Referral Hospital. No study had explored potential DDIs in outpatient settings of Mbarara city. This study aimed to determine the prevalence, severity, and factors associated with potential drug-drug interactions in outpatient settings in Mbarara city.

## Methods

A cross-sectional study was performed in 18 NDA licensed private human pharmacies in Mbarara city. The 18 private pharmacies were selected by random sampling from the NDA list of 84 registered and licensed private human Pharmacies in Mbarara city. This was done by arranging the pharmacies in ascending order of their NDA registration numbers, assigning them numbers from 1 to 84 and then generating 25 Random numbers from 1 to 84 using Microsoft Excel 2021 software. The pharmacies represented by the first 18 random numbers were selected for this study. The extra 7 random numbers were utilized when any of the first 18 selected pharmacies did not consent to conducting this study at their premises. The study was conducted for two consecutive months with the earliest prescriptions assessed dated 2nd August, 2022 and the latest dated 29^th^September, 2022.

The sample size was determined using Kish-Leslie formula for cross-sectional studies which is stated below [16].

$$\textrm{N}=\frac{\textrm{Z}{\upalpha}^2\textrm{P}\left(1-\textrm{P}\right)}{\updelta 2}$$

Where Zα = Standard normal deviate at 95% confidence interval (*P* < 0.05).

P = Estimated prevalence of an indicator δ = Absolute error between the estimated and true population (0.05).

N = Minimum Sample size required.

The value of P was 0.23, given the prevalence of potential DDIs at MRRH that was reported by Lubinga and Uwiduhaye in 2011 [7].

$$\textrm{N}=\frac{1.96^{2\ast }0.23\left(1-0.23\right)}{0.05^2}=273\ \textrm{Prescriptions}$$

We obtained our sample size from 18 selected private pharmacies, considering at least 16 prescriptions from each one of them.

All prescriptions and clinical information from them were collected prospectively as they were presented at the private pharmacies for filling. Consecutive sampling of prescriptions received at the private pharmacies was employed to attain our required sample size. A data collection form which captured patients’ demographics, diagnosis, number of comorbidities, drug regimen and the level of health care facility where the prescription was written was used to extract data from the prescriptions. The prescriptions didn’t note occurrence of any ADR suggestive of drug-drug interaction. The data collection was done by ourselves to ensure authenticity of the data collected. No medication surveillance or patient follow up was conducted and no interaction between the researchers and the patients occurred. Permission was sought from the Pharmacy managers to conduct our study at their premises. Informed consent was obtained from the participants using informed consent forms before data from the prescriptions they presented was collected to be used for our study.

All prescriptions that were valid basing on the presence of the prescriber’s name and signature/stamp plus health facility name and address were included for this study. Prescriptions of only the first visit during our study were considered and no follow-up visit prescriptions were included. This was achieved by writing a unique code number at the bottom right corner of each prescription considered, which was used to identify the prescription in case it was presented on a follow up visit and exclude it. Prescriptions having no drug, having only one drug prescribed, having only topical skin care products prescribed, those that were illegible and all that were written before the month of August 2022 were excluded from the study. Prescriptions with only one drug were excluded because a DDI will only occur between two or more drugs. Most topical skin care products on the other hand have negligible systemic bioavailability and are always intended for local action, thus neither their pharmacokinetics nor pharmacodynamics are affected or affect other drugs in a clinically significant manner. The study protocol was approved by the Ethics committee of the Department of Pharmacy of Mbarara University of Science and technology. All medical data and patient demographic data were collected from the presented prescriptions including sex, age, diagnosis, number of comorbidities, drug regimens prescribed, and level of health care where the prescription was written. Prescriber’s details and patient specific information were not recorded for purposes of confidentiality. The collected data was kept in a file only accessible to the research team members.

All patient prescriptions were scanned for potential DDIs utilizing the Lexicomp drug interactions database version 7.5.4. The identified potential DDIs were further categorized according to risk and severity using the same version of the Lexicomp drug interactions database. The database identifies the risk of drugs interacting, mechanism of drug interaction, severity, reliability rating, and outlines the clinical management of the interaction. Only potential DDIs of clinical significance i.e., risk categories C, D and X according to Lexicomp drug interaction database version 7.5.4 were identified and considered for this study.

The prevalence of potential DDIs was calculated as the ratio of the number of prescriptions with at least one potential DDI to the total number of prescriptions assessed, expressed as a percentage. Bivariate and multivariate logistic regression analysis was performed to identify risk factors associated with the occurrence of clinically significant potential DDIs. The outcome variable was the incidence of at least one clinically significant potential DDI per prescription. At bivariate analysis, we analyzed categorical variables using cross tabulations, Crude Odds Ratios (COR) and chi-square test to assess for the association between patient demographic and clinical characteristics (age, sex, number of comorbidities, number of drugs prescribed, level of health care and therapeutic drug categories prescribed) with the outcome variable. Exposure variables with bivariate *p* values less than 0.2 (age (18-59 yrs), age (≥60 yrs), number of comorbidities, antifungals, antibacterials, antihypertensives, analgesics and corticosteroids) were included in the multivariate logistic regression model. We performed a stepwise and backward selection procedure to determine the final parsimonious model of independent factors associated with our outcome of interest. Confounding and interaction were assessed and the final model checked for goodness of fit using the Hosmer Lemeshow test. The Adjusted Odds Ratios (AOR) and their corresponding *P* values were tabulated. A *P* value <=0.05 was considered to be statistically significant. Statistical data analysis was performed using STATA version 13.

## Results

### Sociodemographic and clinical characteristics of patients

**Fig. 1:**
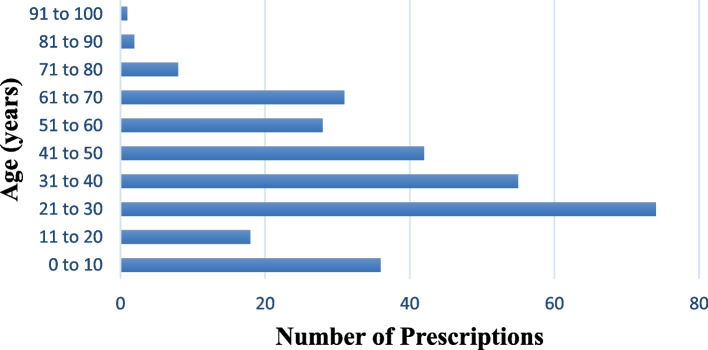
Distribution of the prescriptions among different age-groups

**Fig. 2:**
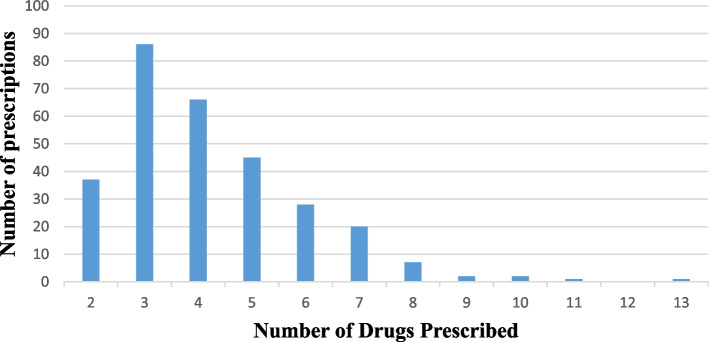
Distribution of the prescriptions among the number of drugs prescribed

In this study we included a total of 295 patient prescriptions, each prescription for a different patient ranging from 0.13 to 93 years with a mean age of 36.49 ± 19.75, presented to 18 community pharmacies in Mbarara city. About half (149, 50.5%) of the patients were females, the majority (199, 67.5%) were adults 18–59 years of age whereas most (208, 70.5%) had a comorbid condition. Over one half (162, 54.9%) of the prescriptions were received from hospitals and majority of the prescriptions had 4 or more drugs prescribed (*n* = 175, 59.32%). Urogenital diseases (*n* = 49, 16.61%) were the most frequently diagnosed followed by gastrointestinal (*n* = 46, 15.59%) and cardiovascular diseases (*n* = 42, 14.24%). These results are summarized in Table 1 below with their corresponding potential DDIs prevalence. Figure 1 shows the age-wise distribution of the prescriptions and Fig. 2 demonstrates distribution of the prescriptions among the number of drugs prescribed. The prescriptions contained 143 unique diagnoses and the commonest included Urinary Tract Infection (*n* = 33, 11.19%), Hypertension (*n* = 28, 9.49%), Diabetes Mellitus (*n* = 17, 5.76%), Peptic Ulcer Disease (*n* = 14, 4.75%), Upper Respiratory Tract Infection (*n* = 10, 3.38%), Malaria (*n* = 7, 2.37%), Typhoid (*n*=7, 2.37%) and Arthritis (*n* = 6, 2.03%).

**Table 1:** Sociodemographic and clinical characteristics of patients

Variables	Category	No. of prescriptions (%)	No. of prescriptions with pDDI (%)
Age	Paediatrics (0–17 years)	45 (15.25)	8 (17.78)
	Adults (18–59 years)	199 (67.46)	75 (37.69)
	Elderly (≥ 60 years)	51 (17.29)	28 (54.90)
Gender	Male	146 (49.49)	58 (39.73)
	Female	149 (50.51)	53 (35.57)
Number of comorbidities	0	208 (70.51)	65 (31.25)
	≥1	87 (29.49)	46 (52.87)
Level of healthcare	Clinic/Medical centre	99 (35.56)	36 (36.36)
	HC II	4 (1.36)	2 (50.00)
	HC III	19 (6.44)	6 (31.58)
	HC IV	11 (3.73)	5 (45.45)
	Hospital	162 (54.92)	62 (38.27)
Number of drugs prescribed	< 4	120 (40.46)	32 (26.67)
	≥4	175 (59.32)	79 (45.14)
Diagnosis/ Disease/ Disorder	Cardiovascular	42 (14.24)	18 (42.86)
	Endocrine	23 (7.80)	20 (86.96)
	ENT	3 (1.02)	2 (66.67)
	Gastrointestinal	46 (15.59)	12 (26.09)
	Hepatic	2 (0.68)	1 (50.00)
	HIV	5 (1.69)	4 (80.00)
	Musculoskeletal	21 (7.12)	11 (52.38)
	Neoplastic	5 (1.69)	3 (60.00)
	Others	54 (18.31)	8 (14.81)
	Psychiatric	12 (4.07)	8 (66.67)
	Reproductive	5 (1.69)	1 (20.00)
	Respiratory	28 (9.49)	8 (28.57)
	Urogenital	49 (16.61)	15 (30.61)

### Drug characteristics

The prescriptions studied had different therapeutic classes of drugs prescribed and these included; antibacterial agents (*n* = 182, 61.69%), analgesic agents (*n* = 98, 33.22%), anti-hypertensive agents (*n* = 55, 18.64%), corticosteroids (*n* = 35, 11.86%), anti-diabetic agents (*n* = 21, 7.12%), anti-fungal agents (*n* = 20, 6.78%), and the others category of drugs (*n* = 187, 63.39%). The classes of drugs that were grouped as others had very low incidence in the prescriptions and these included; anti-coagulants, anti-convulsants, anti-acids, anti-psychotics, anti-epileptics, anti-viral agents, anti-depressants among others.

### Prevalence of potential DDIs

**Fig. 3:**
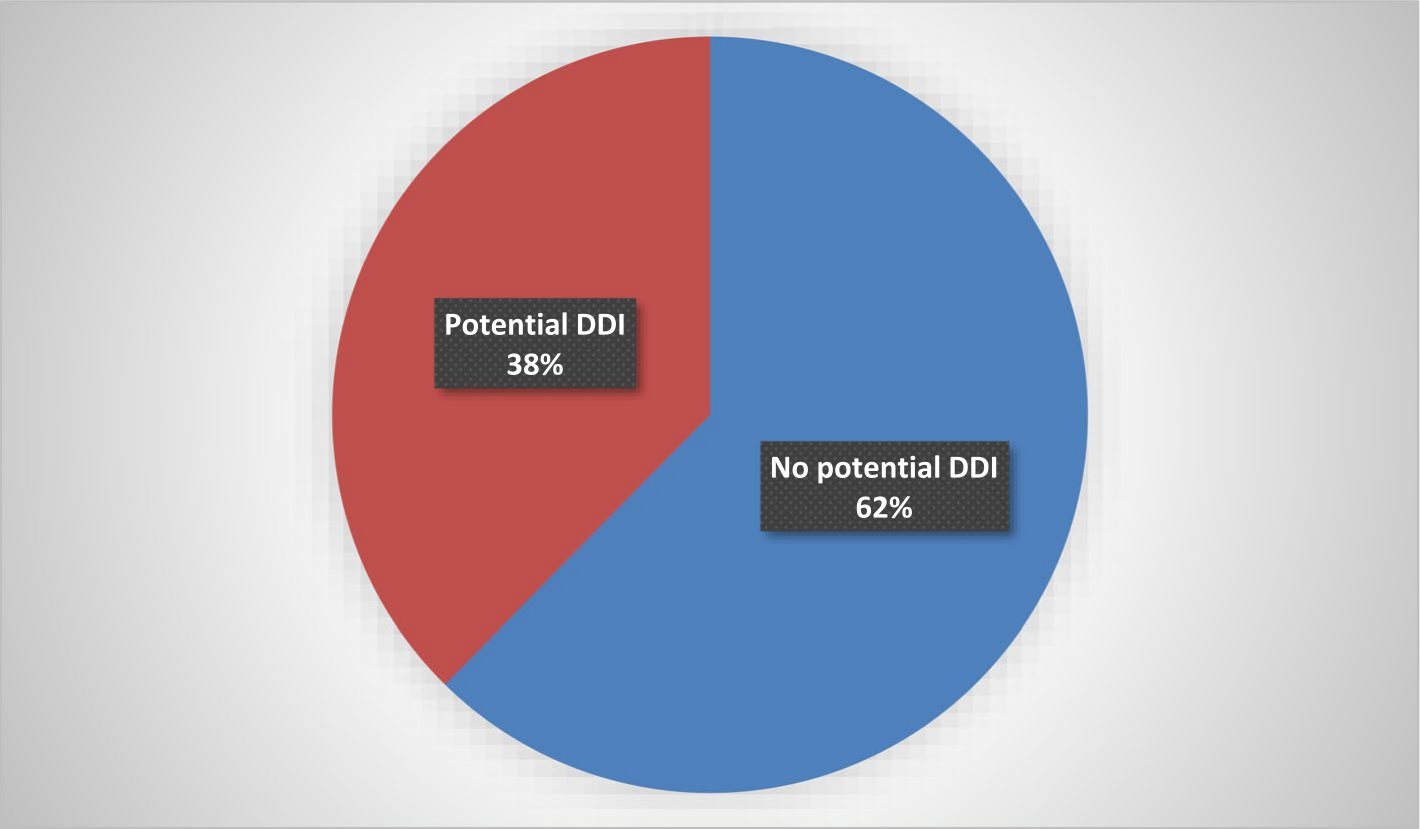
Prevalence of potential DDIs

Out of the 295 prescriptions that were assessed, *n* = 111 (37.6%) had at least one clinically significant potential DDI i.e., potential DDI of risk category C, D or X (Fig. 3). The most frequent drug combinations involved in the potential DDIs are presented in Table 2.

**Table 2:** The most frequent drug combinations involved in the potential DDIs identified

Potentially interacting pair	Frequency	Example	Risk Category	Potential adverse effect	Severity
Anti-diabetics + Hypoglycemia associated agents	21	Metformin + Quinine	C	Hypoglycemia	Moderate
NSAIDS + Quinolones	16	Ibuprofen + Levofloxacin	C	Seizures	Major
Anti-diabetics + Hyperglycemia associated agents	15	Metformin + Furosemide	C	Poor glucose control / Reduced efficacy of anti-diabetic agents	Moderate
NSAIDS + Systemic corticosteroids	14	Diclofenac + Dexamethasone	C	Gastrointestinal bleeding	Moderate
Oral Tetracyclines/ Quinolones + Oral Group II Metallic salts	7	Doxycycline + Ferrous sulphate	D	Treatment failure due to diminished effect of the antibiotic	Moderate to Major
Sulfonylureas + Thiazolidinediones	6	Glimepiride + Pioglitazone	D	Hypoglycemia	Moderate
Opioid agonists + CNS Depressants	5	Tramadol + Gabapentin	D	Slowed or difficult breathing, Respiratory depression	Major
NSAIDS + Rivaroxaban	4	Aceclofenac + Rivaroxaban	D	Excessive bleeding	Major
NSAIDS + Beta blockers	3	Meloxicam + Nebivolol	C	Elevated blood pressure	Moderate

### Frequency and classification of potential DDIs

A total of 216 potential DDIs of different risk categories were identified from the 295 prescriptions that were assessed; category C (*n* = 178, 82.41%) followed by category D (*n* = 34, 15.74%), and category X (*n* = 4, 1.85%).

In severity, most (181, 83.8%) of the DDIs were moderate, followed by major (31, 14.35%) and minor (4, 1.85%) as shown in Table 3. The most severe and highest risk potential DDIs that were identified are presented in Table 4.

**Table 3:** Potential DDI risk and severity categories

Classification	Category	No. (%)
Risk	C	178 (82.41)
	D	34 (15.74)
	X	4 (1.85)
Severity	Minor	4 (1.85)
	Moderate	181 (83.8)
	Major	31 (14.35)

**Table 4:** The most severe and highest risk potential DDIs that were identified

Potential DDI	Potential Duration of concomitant therapy	Risk category	Severity	Potential adverse effects
Doxycycline + Isotretinoin	14 days	X	Major	Intracranial hypertension
Domperidone + Clarithromycin	7 days	X	Major	QTc- prolongation, sudden cardiac death
Celecoxib + Ibuprofen	3 days	X	Major	Gastrointestinal ulceration and bleeding
Spironolactone + Potassium Chloride supplements	7 days	X	Major	Severe hyperkalemia
Rivaroxaban + Aceclofenac	5 days	D	Major	Excessive/Uncontrolled bleeding
Morphine + Pregabalin	30 days	D	Major	Slowed or difficult breathing, Respiratory depression
Tramadol + Gabapentin	5 days	D	Major	Slowed or difficult breathing, Respiratory depression
Dolutegravir + Carbamazepine	60 days	D	Major	Treatment failure due to diminished efficacy of Dolutegravir
Haloperidol + Chlorpromazine	30 days	D	Major	Arrhythmias, Torsades de Pointes
Morphine + Chlorpheniramine	5 days	D	Major	Slowed or difficult breathing, Respiratory depression

### Bi-variate logistic regression

A total of 14 independent variables (factors) were analyzed in bivariate logistic regression. Only 9 out of the 14 factors qualified for multivariate logistic regression analysis having had *P*-values of < 0.2. The factors that qualified for multivariate logistic regression analysis included age (18–59) years, age ≥ 60, number of comorbidities, number of drugs prescribed, presence of antifungals, presence of antibacterials, presence of antihypertensives, presence of analgesics and presence of corticosteroids in a prescription. However, number of drugs prescribed was later excluded from the multivariate logistic regression model because it exhibited collinearity with the number of comorbidities.

### Multivariate logistic regression

The multivariate logistic regression analysis revealed only 5 out of the 8 factors considered for multivariate analysis to be associated with potential DDIs. It showed that patients with one or more comorbidities were about 3 and a half times more likely to have prescriptions with potential DDIs compared to those with no comorbidity. Patients who were prescribed antifungals were 3.69 times more likely to have potential DDIs in their prescriptions compared to those who were not prescribed antifungals. Patients who were prescribed antihypertensives were 3.82 times more likely to have potential DDIs in their prescriptions compared to those who were not prescribed anti-hypertensive medication.

Additionally, patients who were prescribed analgesics were 4.9 times more likely to have potential DDIs in their prescriptions compared to those who were not prescribed analgesics. Finally, the patients who were prescribed corticosteroids were 5.2 times more likely to have potential DDIs in their prescriptions compared to those who were not prescribed corticosteroids. Table 5 summarizes the results from bivariate and multivariate logistic regression analysis of the factors associated with potential drug-drug interactions in prescriptions presented at private pharmacies in Mbarara city.

**Table 5:** Bi-variate and multivariate analysis of factors associated with the occurrence of potential DDIs

Exposure Variables	Category	Outcome (Presence of pDDI)		Crude Odds Ratios (95% CI)	*P* value	Adjusted Odds Ratios (95% CI)	*P* value
		Yes	No				
Gender	Female	53	96	ref			
	Male	58	88	1.19 (0.74,1.91)	0.462		
Age	< 18 yrs	8	37	ref			
	18–59 yrs	75	124	2.80 (1.24,6.33)	**0.014**	1.67 (0.68,4.11)	0.268
	≥ 60 yrs	28	23	5.63 (2.19,14.45)	**< 0.001**	2.64 (0.89,7.84)	0.080
Level of Health care	Clinic	36	63	ref			
	HC II	2	2	1.75 (0.24,12.96)	0.584		
	HC III	6	13	0.81 (0.28,2.31)	0.690		
	HC IV	5	6	1.46 (0.42,5.12)	0.556		
	Hospital	62	100	1.09 (0.65,1.82)	0.757		
No. Of Comorbidities	0	65	143	ref			
	≥1	46	41	2.45 (1.48,4.12)	**0.001**	3.47 (1.86,6.47)	**< 0.001**
No. Of drugs prescribed	< 4	32	88	ref			
	≥4	79	96	2.81 (1.37,3.74)	**0.001**		
Anti-fungal	No	99	176	ref			
	Yes	12	8	2.6 (1.05,6.74)	**0.038**	3.69 (1.31,10.36)	**0.013**
Antibacterials	No	53	60	ref			
	Yes	58	124	0.53 (0.33,0.86)	**0.01**	0.73 (0.38,1.39)	0.342
Anti-hypertensive	No	79	161	ref			
	Yes	32	23	2.84 (1.56,5.17)	**0.001**	3.82 (1.93,7.58)	**< 0.001**
Analgesics	No	56	141	ref			
	Yes	55	43	3.22 (1.94,5.34)	**< 0.001**	4.90 (2.68,8.95)	**< 0.001**
Corticosteroids	No	88	172	ref			
	Yes	23	12	3.75 (1.78,7.88)	**0.001**	5.20 (2.25,12.00)	**< 0.001**
Anticonvulsants	No	99	169	ref			
	Yes	12	15	1.37 (0.61,3.04)	0.444		
Minerals and Vitamins	No	91	152	ref			
	Yes	20	32	1.04 (0.56,1.93)	0.891		
Anti-acids	No	93	149	ref			
	Yes	18	35	0.82 (0.44,1.54)	0.544		
Others	No	67	120	ref			
	Yes	44	64	1.23 (0.76,2.00)	0.402		

## Discussion

### Prevalence of potential DDIs

In this study, we obtained 295 prescriptions from 18 private pharmacies in Mbarara city, each contributing at least 16 prescriptions which we considered for analysis. We found that the prevalence of potential drug-drug interactions was 37.64%. This prevalence is comparable with 34.4% that was reported by a study in Romania [17].

The current prevalence, however, is considerably higher than 22.65% that was reported by a large cohort study of older community-dwelling adults in Kenya [18]. This could be because the study considered only drug-drug interactions involving cardiovascular and CNS drugs leaving out other drug categories. The study also considered potential DDIs among only older adults with a mean age of 78 ± 5.53, unlike our study which considered potential DDIs among all age groups, with a mean age of 36.49 ± 19.75. Our result of 37.64% prevalence of potential drug-drug interactions is also higher compared to 18.7% reported from a study in patients attending a specialist HIV outpatient clinic in Kampala, Uganda [19]. The difference in prevalence here is probably due to the fact that the study only considered DDIs involving ART drugs leaving out other drug categories. With a 37.64% prevalence of potential DDIs, approximately 38 out of every 100 patients filling their prescriptions at private pharmacies in Mbarara city are likely to experience more frequent adverse drug reactions resulting from the DDIs. These lower their quality of life and hinder their adherence and compliance to medication. They are also likely to experience therapeutic failure which may necessitate and /or lengthen hospitalization, consequently expanding the cost of treatment [12]. There is therefore urgent need for prescribers to implement strategies that minimize the incidence of potential DDIs. Such strategies include but are not limited to carrying out comprehensive review of the current medications a patient is taking on any clinical visit, carrying out medication reconciliation during transition of care, use of electronic health record systems to aid tracing and identification of potential DDIs and utilization of specialized software or databases like Lexicomp to screen for potential DDIs. In real life conditions, an important question is whether busy clinicians will be willing and able to devote their time to tracing all possible potential DDIs. In light of this question, pharmaceutical care is an appropriate alternative for the success of the strategies. With their specific knowledge and general availability, pharmacists have the capacity to track and manage potential DDIs. In conjunction with electronic health systems and specific tools, they will greatly contribute to management of DDIs and ensure patient safety [11].

### Risk categories of potential DDIs

The risk categories describe clinical relevance/significance and intensity of clinical actions required to prevent adverse effects of the potential DDIs. Lexicomp classifies risk categories as A, B, C, D and X. The shift from A to X corresponds to an increase in intensity of clinical actions that should be taken. The risk categories are detailed in Table 6 below.

**Table 6:** Description of potential DDI risk categories [20, 21]

Risk category	Description
**A**	Not clinically significant. Research data have not demonstrated interaction between the specified agents.
**B**	Not clinically significant. There is evidence for possible interaction but little to no evidence of clinical concern resulting from concomitant use of the specified agents.
**C**	Clinically significant. The benefits of concomitant use of the specified agents often outweigh the risks. An appropriate monitoring plan is required to identify potential negative effects of the interaction and dosage adjustments of one or both agents involved may be needed in some patients
**D**	Clinically significant. A patient-specific assessment should be conducted to determine whether the benefits of concomitant therapy outweigh the risks. Specific actions should be taken in order to realize the benefits and/or minimize the risks i.e., aggressive monitoring, empiric dosage changes, or choosing alternative agents
**X**	Clinically significant. Concomitant use of the specified agents should be avoided because the risks involved always outweigh the benefits

This study only considered interactions of risk category C, D and X which are described as being clinically relevant.

Of the 216 potential DDIs identified, 82.41% were of risk category C, 15.74% of risk category D and 1.85% of risk category X. These results show that majority of the potential DDIs that were identified required no intense clinical action but just monitoring. However, in an outpatient setting, clinical monitoring is very limited and ineffective. Majority of the identified potential DDIs (category C) would manifest among outpatients with their undesirable effects due to absence of or inadequate monitoring, consequently worsening patient outcomes. The category D interactions require aggressive monitoring which is not feasible in outpatient settings. Patients have increased risk of severe outcomes like advanced disease and life-threatening adverse drug reactions. Majority of the category X potential DDIs are severe DDIs with a high risk of mortality if not identified and managed timely and appropriately.

### Severity of potential DDIs

Severity categories describe the impact of the DDIs on the patient’s condition/health. Basing on severity, the DDIs are categorized as minor, moderate and major. Minor severity refers to an interaction that is bothersome, but otherwise not medically detrimental. Moderate severity indicates that the patient’s condition may deteriorate due to the interaction, requiring additional care or extended hospitalization. Major severity indicates that the interaction may be life-threatening or cause permanent damage [20].

We found that DDIs of moderate severity (83.8%) were the most prevalent. This finding is comparable to the results of a number of previous studies that were done in Jordan, Pakistan, Switzerland and Ethiopia among others, with different study populations and settings from our study [8, 22–25].. This reinforces the urgent need to identify and prevent occurrence of the clinically significant potential DDIs because majority of them may deteriorate the patient’s condition, necessitate additional healthcare and /or lengthen hospitalization consequently raising healthcare costs. If not managed appropriately, they definitely have the potential to increase morbidity and mortality of the patients. When antibiotics are affected by moderate or major drug interactions, in addition to the probable treatment failure, there is also a significant risk of contributing to antimicrobial resistance. On the other hand, patients may experience adverse drug events from unexpectedly increased serum level of their medications as a result of moderate or major DDIs.

### Factors associated with potential DDIs

Knowledge of factors associated with potential drug-drug interactions is of great importance in healthcare systems. It enables prescribers and other members of healthcare teams to predict, identify, and minimize their occurrence, thus reduce the adverse outcomes.

In our study, the factors that were significantly associated with presence of potential DDIs included having one or more comorbid conditions and taking any of analgesics, antifungals, antihypertensives or corticosteroids concurrently with other drugs. Patients with at least one chronic comorbid condition were about 3.47 times more likely to incur potential drug-drug interaction compared to those without comorbidity. This is in agreement with findings of a prospective study conducted in Bangalore. Another study conducted in India also reported an association between number of comorbidities and potential drug-drug interactions [26, 27].

Our study revealed that prescriptions that contained analgesics were 4.9 times more likely to have a potential DDI compared to those that didn’t contain an analgesic prescribed. Studies done by Moore et al. (2015) and Kardas et al. (2021) specifically considering potential DDIs of analgesic drugs support the positive correlation between prescription of analgesics and the presence of potential DDIs [28, 29].

Our study findings showed that prescriptions that contained anti-fungal agents concomitantly prescribed with other agents were 3.7 times more likely to have a potential DDI compared to those that did not contain any anti-fungal agent prescribed. This finding agrees with the results of two studies conducted in haematological patients in 2014 and 2017 [30, 31].

Our study also detected a positive correlation between the prescription of antihypertensives and presence of potential DDIs, with the prescriptions containing anti-hypertensive agents being 3.82 times more likely to have a potential DDI compared to those without antihypertensives. Similarly, a longitudinal analysis of anti-hypertensive drug interactions in a Medicaid population study reported a positive correlation between the prescription of antihypertensives and presence of potential DDIs [32].

Prescription of corticosteroids was also significantly associated with potential drug-drug interactions. Prescriptions containing corticosteroids were 5 times more likely to contain potential DDIs compared to those that had no corticosteroids prescribed. There are very few previous studies that considered analysis of the association between corticosteroids and potential DDIs, among which is a recent study about the risks of potential DDIs in COVID-19 patients treated with corticosteroids [33]. This study reported that the addition of corticosteroids to background therapies resulted in dramatic increase in the number of DDIs classified as moderate, which is similar to our study findings. In light of the above findings, prescribers should be more vigilant when prescribing drug categories including analgesics, antifungals, antihypertensives and corticosteroids so as to minimize potential DDIs.

### Strength of the study

This study included prescriptions of patients of all ages, all disease categories, and from all levels of healthcare. This allowed us to asses for the association of a wider range of factors with potential DDIs in the outpatient setting which most previous individual studies did not analyse.

## Limitations of the study

Our study was solely conducted in an urban setting and thus, lacks generalizability to the general population in the region as well as to other health care settings in other regions in Uganda. The other limitation was inability to follow-up the patients with potential drug-drug interactions for adverse drug events. This was because the study was retrospectively conducted on prescription notes without directly interacting with patients.

## Conclusion

The prevalence of potential DDIs in prescriptions received by private pharmacies in Mbarara city was high with majority of them being moderate in severity. Having 1 or more comorbidities and prescribing of therapeutic drug categories including antifungals, antihypertensives, analgesics, or corticosteroids concurrently with other drugs were significantly associated with potential DDIs. Prescribers should be vigilant while prescribing for patients with multimorbidity or during ordering any agent from these drug classes, to prevent subsequent adverse drug events, improve patient outcomes and minimize health care costs.

## Recommendations

Based on the findings of our study, we recommend that the Ministry of Health and management of private healthcare facilities should provide continuous education and training programs for health care professionals to enhance their knowledge about drug interactions, including the knowledge of potential risks, common scenarios and how to use available DDIs identification tools like Lexicomp database. They should adopt and promote the use of electronic health service and record systems to ease medication reconciliation during transition of care, and aid tracking and identification of potential DDIs. They should also employ more pharmacists to work with prescribers in identifying and preventing DDIs. Pharmacists and prescribers should allocate adequate time to medication review processes to foster effective potential DDIs identification and resolution. They should educate patients about the importance of telling their health care providers all the medications they are taking including over-the-counter drugs, vitamins and herbal supplements.

Prescribers should be more vigilant to minimize potential DDIs whenever they prescribe drug categories including analgesics, antifungals, antihypertensives and corticosteroids.

The Ministry of Health should fund further research to assess potential DDIs in rural areas to avail information that will guide service delivery to rural areas with regards to prevention of adverse effects of potential DDIs at national level.

## Data Availability

The data that support the findings of this study are available on request from the corresponding author. The data are not publicly available due to patient privacy or ethical restrictions.

## Abbreviations

ADR: Adverse Drug Reaction
ART: Antiretroviral therapy
CI: Confidence Interval
CNS: Central Nervous System
COVID-19: Corona Virus Disease of 2019
DDIs: Drug-Drug Interactions
ENT: Ear Nose and Throat
HC e.g., HCIII: Health Centre III (three)
HIV: Human Immunodeficiency Virus
NDA: National Drug Authority
pDDI: potential Drug-Drug Interactions.
